# The Intriguing Role of TLR Accessory Molecules in Cardiovascular Health and Disease

**DOI:** 10.3389/fcvm.2022.820962

**Published:** 2022-02-14

**Authors:** Taisiya Bezhaeva, Jacco Karper, Paul H. A. Quax, Margreet R. de Vries

**Affiliations:** ^1^Department of Surgery and Einthoven Laboratory for Experimental Vascular Medicine, Leiden University Medical Center, Leiden, Netherlands; ^2^Department of Cardiology, Wilhelmina Hospital Assen, Assen, Netherlands

**Keywords:** vascular remodeling, TLR signaling, TLR accessory molecules, inflammation, cardiovascular disease, myocardial infarction, NF-kappa B

## Abstract

Activation of Toll like receptors (TLR) plays an important role in cardiovascular disease development, progression and outcomes. Complex TLR mediated signaling affects vascular and cardiac function including tissue remodeling and repair. Being central components of both innate and adaptive arms of the immune system, TLRs interact as pattern recognition receptors with a series of exogenous ligands and endogenous molecules or so-called danger associated molecular patterns (DAMPs) that are released upon tissue injury and cellular stress. Besides immune cells, a number of structural cells within the cardiovascular system, including endothelial cells, smooth muscle cells, fibroblasts and cardiac myocytes express TLRs and are able to release or sense DAMPs. Local activation of TLR-mediated signaling cascade induces cardiovascular tissue repair but in a presence of constant stimuli can overshoot and cause chronic inflammation and tissue damage. TLR accessory molecules are essential in guiding and dampening these responses toward an adequate reaction. Furthermore, accessory molecules assure specific and exclusive TLR-mediated signal transduction for distinct cells and pathways involved in the pathogenesis of cardiovascular diseases. Although much has been learned about TLRs activation in cardiovascular remodeling, the exact role of TLR accessory molecules is not entirely understood. Deeper understanding of the role of TLR accessory molecules in cardiovascular system may open therapeutic avenues aiming at manipulation of inflammatory response in cardiovascular disease. The present review outlines accessory molecules for membrane TLRs that are involved in cardiovascular disease progression. We first summarize the up-to-date knowledge on TLR signaling focusing on membrane TLRs and their ligands that play a key role in cardiovascular system. We then survey the current evidence of the contribution of TLRs accessory molecules in vascular and cardiac remodeling including myocardial infarction, heart failure, stroke, atherosclerosis, vein graft disease and arterio-venous fistula failure.

## Pattern Recognition Receptors in Cardiovascular Disease

Upon cardiovascular tissue injury a number of self-derived immunomodulatory molecules are released into the systemic circulation and the interstitial space where they act as damage associated molecular patterns (DAMPs) also called alarmins. Together with exogenous pathogen-associated molecular patterns (PAMPs), DAMPs are recognized by highly specific germline encoded pattern recognition receptors (PRRs) to activate various immune signaling cascades (1).

Depending on cell localization PRRs can be divided into two major groups, the transmembrane protein families—Toll-like receptors (TLRs) and C-type lectin receptors (CLRs), and the cytoplasmic protein families—nucleotide-binding oligomerization domain (NOD-like) receptors (NLRs), retinoic acid-inducible gene-I-like receptors (RLRs) and absent in melanoma-2 (AIM2)-like receptors (ALRs) (2). There are several classes of PRRs known to accelerate the inflammatory response in cardiovascular disease (CVD) particularly when the heart or vessel wall respond to ischemia or mechanical stress (3–6). The classic PRRs, TLRs and the more recently discovered NLRs, interact with each other to facilitate progression of several CVDs (e.g., atherosclerosis and heart failure) (4, 7).

Although mainly expressed on immune cells, PRRs are present on cardiovascular cells including endothelial cells, cardiomyocytes, smooth muscle cells (SMCs) and fibroblasts where they trigger a wide array of immune responses against cell damage (8, 9).

TLRs are the first discovered and most essential PRR (10). Initially it was described as a receptor with similarity to the Drosophila Toll protein, which was originally identified in fly embryonic development. Toll was shown to be critical for Drosophila immune defense against pathogens via induction of pathways homologous to those activating the transcription factor Nuclear Factor kappa-light-chain-enhancer of activated B cells (NF-κB) (11). TLRs can also recognize non-microbial endogenous molecules that are released upon cell death or present in the extracellular matrix (12, 13). TLR-ligand interaction leads to the activation of both innate and adaptive immune responses, culminating in activation of transcription factors and subsequent production of pro-inflammatory cytokines and type I interferons. These downstream pathways include positive feedback loops which can culminate in a strong response that can induce repair of tissue damage but can overshoot and with that cause acute and chronic inflammation such as atherosclerosis. Especially, accessory molecules can guide and dampen these responses toward an adequate response.

NLRs are intracellular sensors of DAMPs that can be divided into 4 subfamilies depending on the configuration of N-terminal domain. They orchestrate a number of pathways including NF-κB signaling, retinoic acid–inducible gene-I–like receptor signaling, autophagy, major histocompatibility complex gene regulation, reproduction, and development. NLRP3 and NOD1 gathered specific attention in the field of CVDs due to their association with inflammasomes (14, 15). NLRP3 and NOD1 inflammasomes play an essential role in atherogenesis (16–19), aortic aneurysm formation (20), cardiac inflammation and fibrosis (21–24). Recently, AIM2 inflammasome was shown to recognize cytoplasmic self-double-stranded DNA which might point toward a role in sterile inflammation (25).

CLRs are increasingly recognized as PRRs that are not only important in host defense against pathogens but also can recognize number of DAMPs in the progression of cancer, CVD and autoimmune diseases (26, 27). It is a large family of more than 1,000 proteins that have been placed into 17 groups based on their structure and/or function. One of the subgroups of CLRs is Dectin-1 cluster that is comprised of seven receptors and draw particular attention to its role in CVD. For instance, LOX-1 has been extensively studied in atherosclerosis progression and associated hypertension and stroke (28, 29). For the scope of this review it is important to mention that CLR signaling pathways in some cases can synergize with TLR signaling pathways to upregulate cytokine and chemokine production.

In further sections of this review, we will discuss key characteristics of TLRs, with a particular focus of the role of TLRs and their accessory molecules in the development of cardiovascular-related pathologies.

## TLR Family and Pathway Signaling

The TLR family is a key component of the innate immune system that constitutes of 13 different receptors that are evolutionarily conserved between species. Humans express 10 functional TLRs (TLR1 to TLR10) whereas mice express 12 (TLR1–TLR9, 11, 12, and 13). Except for speculative ligands for TLR10, most of other TLRs-ligand pairs are known. Ligands recognized by TLRs include lipids, lipoproteins, proteins and nucleic acids derived from a wide range of exogenous and endogenous sources.

**Figure 1:**
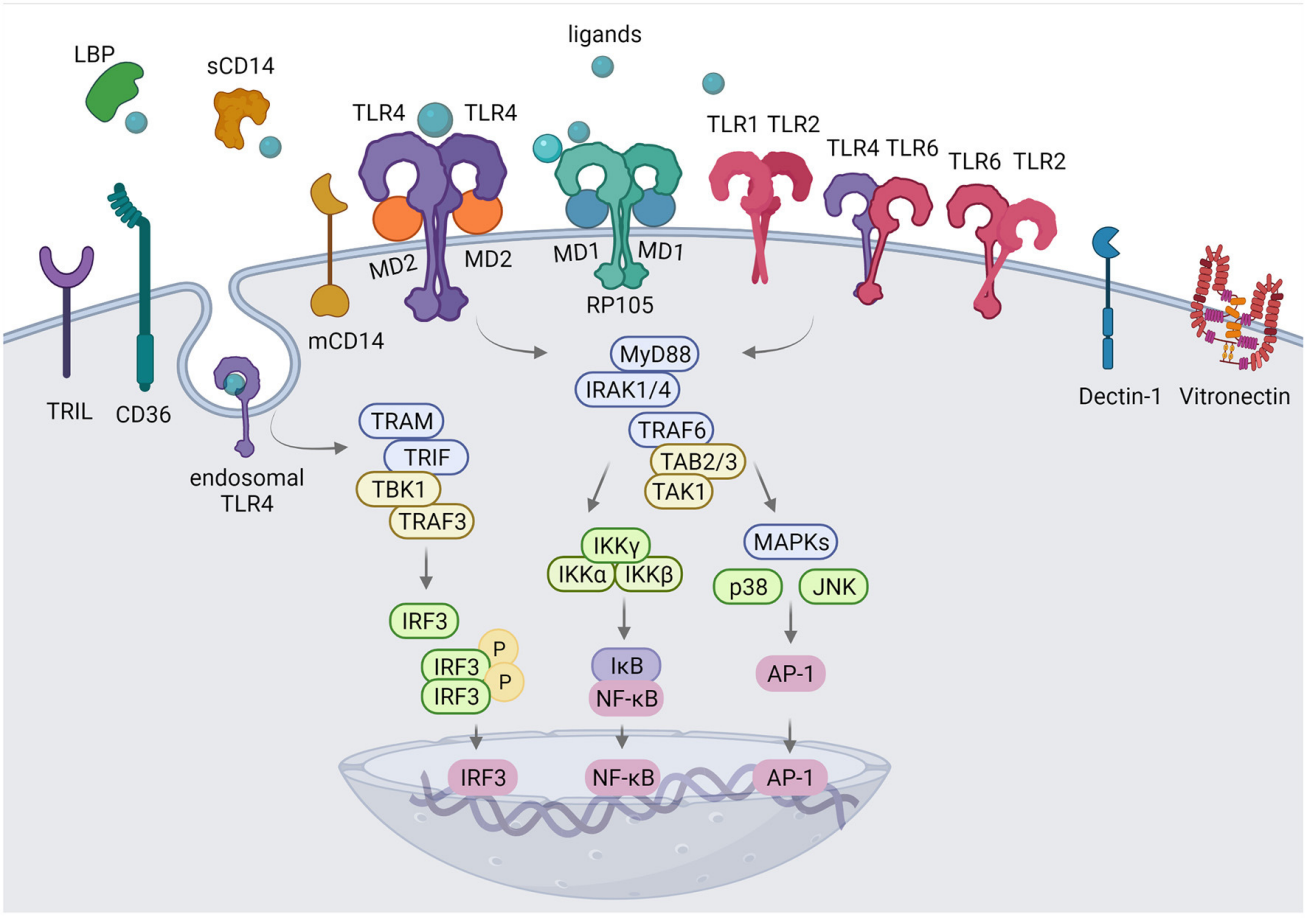
Membrane TLRs and their accessory molecules. Cell surface TLR1, TLR2, TLR4, and TLR6 are essential for the recognition of exogenous and endogenous ligands. TLR1/2, TLR2/6 heterodimers and TLR4/TLR4 homodimer utilize MyD88-dependent pathway to control inflammatory responses *via* activation of NF-κB and AP-1 transcription factors, endosomal TLR4 activates TRAM/TRIF-dependent pathway resulting in type I IFN responses. CD36 induces the assembly of the TLR4/6 and TLR2/6 heterodimers. CD14 can be secreted as a soluble molecule (sCD14) or a membrane bound protein (mCD14) and is involved in ligand delivery to several TLRs. LPS-binding protein (LBP) binds to lipopolysaccharide (LPS) and presents it to CD14. MD2 is necessary for TLR4 to bind to LPS and homodimerize. RP105-MD1 complex has a structural similarity to TLR4-MD2 and exerts dual regulatory activity on TLR4 and TLR2 -regulated inflammatory response. Dectin-1 facilitates TLR2 signaling whereas TRIL interacts with ligands to activate TLR4 signaling. Vitronectin enhances TLR2 and TLR4-mediated responses. Created with BioRender.com.

TLRs are type I transmembrane receptors that contain a N-terminal ligand recognition ectodomain with signature leucine-rich repeats (LRRs), a single transmembrane helix, and a Toll/interleukin-1 receptor-like (TIR) signaling domain. For a proper signal transduction TLRs form homo- or heterodimers. Subsequently, they can interact with various adaptor proteins, including myeloid differentiation primary response protein 88 (MyD88) and TIR domain containing adaptor protein-inducing interferon IFN-β (TRIF), which leads to downstream activation of MAPKs and activation of transcription factors such as NF-κB, activator protein-1 (AP-1) and interferon regulatory factors (IRFs) (Figure 1). After translocation to the nucleus the transcription factors can induce transcription of proinflammatory genes and interferons.

Depending on the cellular localization and respective ligands TLRs are divided into two subclasses:

(i) Cell surface TLRs (TLR1, TLR2, TLR4, TLR5, TLR6, and TLR11) that recognize exogenous microbial membrane components such as lipids, lipoproteins and proteins and number of endogenous molecules amongst others cell-derived proteins, components and degradation products of the extracellular matrix (ECM), lipoproteins and free fatty acids.

(ii) Intracellular TLRs (TLR3, TLR7, TLR8 and TLR9) that are expressed within the endoplasmic reticulum (ER), endosomes, lysosomes and endolysosomes, and recognize components of nucleic acids (Table 1).

**Table 1:** TLRs and their ligands.

**TLR**	**Exogenous Ligands**	**Endogenous Ligands**
**Localization: Cell surface**		
TLR1	Cooperates with TLR2 to recognize bacterial lipopeptides	Cooperates with TLR2 to recognize amyloids
TLR2	Bacterial lipoproteins, peptidoglycans, lipoteichoic acid, yeast mannans	HSP60, 70, 96; biglycan; HMGB1; hyaluronic acid fragments; human β-defensins; acute serum amyloid A; histones; ECM; serum amyloid A, snapin A; endoplasmin; monosodium urate crystals
TLR4	Bacterial LPS, plant taxol, viral fusian protein	HSP20, 60, 70, 72, 96; biglycan, HMGB1, hyaluronic acid fragments; oxidized LDL; mmLDL, fetuin-A; Ang II; serum amyloid A; histones; S100 proteins; fibronectin; fibrinogen; heparan sulfate; syndecan-1; resistin; β-defensin; surfactant protein A
TLR5	Bacterial flagellin	Flagellin from gut microbiota
TLR6	Cooperates with TLR2 to recognize bacterial lipopeptides, fungal zymozan, modulin	Cooperates with TLR2 and TLR4 to recognize HMGB1; HSPs; ECM; HSP60, −70, −96
TLR10	NA	NA
**Localization: Intracellular**		
TLR3	Viral dsRNA, polyinosine-polycytidylic acid	mRNA
TLR7	Viral ssRNA	Self-ssRNA
TLR8	Viral ssRNA	Self-ssRNA
TLR9	Bacterial and viral DNA	Self-dsDNA; histones; mitochondrial DNA; chromatin immune complexes

Upon internalization the cell surface TLR4 can also localize intracellularly into endosomes. Accessory molecule CD14 assures its proper internalization and switch in signaling pathway toward activation of TRIF-dependent signaling and type-I interferons production (30).

There is a wide variety of ligands recognized by TLRs. But how TLRs are able to discriminate between ligands, self-vs. non-self and orchestrate proper cellular response? The LRRs within ectodomain that are composed of just 20 to 30 amino acids (AA) determine structural confirmation of TLRs for a specific ligand interaction, yet variations in AA sequences are limited (31). TLR accessory molecules serve as another important mechanism to ensure proper detection of DAMPs by specific TLRs.

The classical example of the importance of accessory molecules is TLR4 mediated response to bacterial lipopolysaccharide (LPS). There are a number of key-accessory molecules [LPS-binding protein (LBP), CD14, Myeloid differentiation factor 2 (MD2)] that are required for successful propagation of TLR4 signaling. In the presence of bacterial infection first, soluble plasma protein LBP binds to LPS whereupon it is recognized by CD14—a glycosylphosphatidylinositol-linked, LRRs-containing protein that delivers LBP-LPS complex to the cell surface. On the cell surface TLR4 forms a complex with MD2 that serves as the main LPS-binding site. The resultant formation of a receptor multimer, composed of two copies of the TLR4-MD2-LPS complex, initiates signal transduction by recruiting intracellular adaptor molecules.

## From TLR Ligand Recognition to Signal Transduction in CVD

TLRs and their accessory molecules are present on most of the cells of cardiovascular system including endothelial cells, ***smooth muscle cells***, cardiomyocytes, fibroblasts and resident tissue macrophages (32). A number of DAMPs that are released upon cardiovascular tissue injury serve as TLR ligands to induce nuclear translocation of various transcription factors (e.g., NF-κB) and promote pro-inflammatory cytokine release. TLR-DAMP interactions may exhibit both protective and detrimental effects. A short-term TLR mediated inflammatory response is required for a proper cardiovascular adaptation to stress, and is essential for tissue repair and regeneration, whereas long term or excessive TLR activation induces a chronic inflammatory state resulting in adverse cardiac and vascular remodeling. Thorough data supports the importance of TLRs in the pathogenesis of atherosclerosis (33–35), vein graft disease (9, 36), myocardial infarction (MI) (37, 38), ischemia reperfusion injury (IRI) (39), and cardiac allograft rejection (40).

## TLR Ligands

Amongst the TLRs, membrane bound TLR1, TLR2, TLR4, and TLR6 can be activated by numerous intracellular proteins that are released upon cell damage and cell death [e.g., Heat Shock Proteins (HSPs), High Mobility Group Box-1 (HMGB-1), ATP, mtDNA, RNA and histones]. The intracellular TLR3, TLR7, TLR8, and TLR9 on the other hand are activated by endogenous nucleic acids in endosomes. The role of cell surface TLR5 that recognizes bacterial flagellin was recently depicted in obesity and metabolic syndrome, as it can sense components from gut microbiota to drive systemic inflammation (41, 42). Unlike other TLRs, TLR10 is the unique receptor with anti-inflammatory properties, yet its ligands and functions are not well-defined (43, 44). Besides intracellular proteins, TLRs recognize a number of ECM components [e.g., biglycan, hyaluronic acid, versican, extradomain A of fibronectin (EDA), fibrinogen fragments and surfactant protein A, amyloid-b] and other fragments amongst others including oxidized low density lipoprotein (LDL), free fatty acids (FFA), angiotensin II (AngII), mtDNA, circular RNA, extracellular ATPs and immune complex-containing self-antigens.

A number of DAMPs contribute to cardiovascular remodeling and have been extensively studied in both experimental models and in humans. Following ischemia-reperfusion injury 3 weeks after left coronary artery ligation in rats, administration of HMGB-1 resulted in modulation of inflammation via reduction in dendritic cells, attenuated fibrosis and overall improvement of cardiac function (45). In a similar model, inhibition of HMGB-1 by neutralizing antibodies resulted in enlarged infarct size, increase the plasma troponin-T and norepinephrine content in the heart as compared to untreated animals (46). HMGB-1 also plays a pivotal role in ischemic stroke (47). High levels of systemic HGMB-1 were measured in serums of patients with cerebral ischemia (48). In another study in 338 patients, plasma levels of HMGB-1 was an independent predictor of 1-year clinical outcomes of ischemic stroke (49). A role of HSP60-induced apoptosis via TLR4 in myocyte loss in heart failure was demonstrated on rat primary isolated cardiomyocytes *in vitro* (50). In a prospective study with 251 patients admitted for ST-segment elevation myocardial infarction (STEMI) increase in HSP70 levels were associated with larger infarcts, increased LV dilation and worse clinical outcome post MI (51). Levels of extracellular matrix components, fibronectin, hyaluronic acid and proteoglycans associate with adverse cardiac remodeling post MI. Permanent ligation of the left anterior coronary artery in fibronectin-EDA deficient mice was characterized by reduced inflammation, fibrosis and MMP-2,−9 activity as compared to the WT animals. Together with the reduced recruitment of monocytes and decrease in monocytic TLR2 and CD49 mRNA expression levels after infarction, fibronectin-EDA plays a critical role in adverse cardiac remodeling (52). Another component of ECM—hyaluronan—serves as a promising biomarker for myocardial damage among patients with acute myocardial infarction (AMI). In 56 patients plasma levels of hyaluronan were significantly elevated 30 day after AMI (53). Both fibronectin and hyaluronan were linked to cardiac allograft rejection as demonstrated in heart allografts in rats. Fibronectin protein levels were upregulated in the vessels exhibiting cardiac allograft vasculopathy and in fibrotic areas whereas increased accumulation of hyaluronan was evident in the edematous interstitial tissue in the heart that was infiltrated with lymphocytes (54, 55). During vascular remodeling in vein graft disease events of distension injury lead to endothelial and smooth muscle cell damage, and degradation of ECM in the media and adventitia. Furthermore, the ischemia-reperfusion injury during and after surgery will also result in the production of DAMPs. Release of hyaluronic acid, proteoglycans and fibronectin that act as endogenous TLRs ligands prime proinflammatory responses which further damage vascular cells (56, 57). Another well-described set of DAMPs – lipoproteins and FFA are well-known factors contributing to atherogenesis and are widely used in clinic as prognostic biomarkers for coronary artery disease (CAD). Levels of oxidized low-density lipoprotein were strongly associated with CAD in a cohort of 504 patients whereas in a prospective cohort of 3,315 participants levels of FFA independently predicted all-cause mortality (58, 59). Mitochondrial DNA and circulating extracellular RNA that are released as a consequence of cell death during myocardial IRI can act as DAMPs to induce pro-coagulation and pro-inflammatory responses (60).

Targeting the DAMP/TLR mediated inflammatory response was proven to be successful in small and large animal models, yet clinical translation remains to be very challenging, since the most investigated systemic therapies can give detrimental side effects. In this light fine-tuning of TLR signaling via accessory molecules might provide better therapeutic outcomes.

## TLR Signaling

Despite the wide range of DAMPs recognized by TLRs, their structural organization with extracellular and ligand-binding domains is very similar. Following ligand recognition TLRs will form dimers to trigger recruitment of adaptor proteins MyD88 and TRIF to initiate intracellular signaling. TLR2 will form heterodimers with either TLR1 or TLR6, TLR4 will interact with its accessory molecule MD2 to form homo or heterodimers with TLR6, whereas TLR3 forms homodimers upon dsRNA binding (61).

Activation of the downstream MAPKs and IκB kinase (IKK), resulting in activation of the transcription factors AP-1 and NF-κB, respectively culminates in inflammatory cytokine release. In addition, TRIF recruits another cellular kinase, TANK binding kinase 1 (TBK1), to activate the IRF3 and IFN-I production (62) (Figure 1).

Although the ligand-induced dimerization of TLRs has many common features, the nature of the interactions of the TLR extracellular domains with their ligands varies markedly between TLR paralogs. Accessory molecules play essential role not only in assuring proper TLR/ligand interaction but governing complex TLR signaling.

## Cell Surface Accessory Molecules in Cardiovascular Disease Progression

As discussed earlier in this review, accessory molecules provide mechanisms that can support complexity and diversity of TLR ligand composition. They contribute not only to TLRs signal propagation but facilitate the crosstalk between different TLRs and serve as cofactors.

Delivery of DAMPs by accessory molecules to specific TLRs will assure its proper dimerization, folding, cell localization, and protein processing, all of which guarantee that TLR/ligand interaction will initiate signaling cascades.

Depending on cellular localization, properties and functions TLRs accessory molecules form different groups.

1. TLR accessory molecules that act as cofactors required for cell surface ligand recognition and delivery (LBP, MD2, CD36, CD14, TRIL)
2. TLR accessory molecules that are required for endosomal ligand recognition and delivery (Granulin, HMGB1, LL37)
3. TLR accessory molecules that act as chaperones (Gp96, PRAT4A), trafficking proteins (UNC93B1, AP3) and processing factors (cathepsins, AEP)
4. Adaptor proteins that are required for TLR signaling (MYD88, TRIF, MAL, TRAM)
5. Cross-talk molecules that facilitate inflammatory signal transduction (NOD1, NOD2, NALPs)
6. Proteins with both TLR crosstalk and cofactor function (RP105-MD1, Dectin-1, Vimentin)
7. Receptors that interact with TLRs and passively modulate TLR functions: B cell receptor, RAGE

To restrict the focus of this review, in this review we will only discuss accessory molecules that are required for TLR ligand recognition and delivery on cell surface or involved in crosstalk of signaling pathways (Figure 1). Factors that serve as scaffolding or adaptor proteins required for signaling are excellently reviewed elsewhere (10).

The contribution of inflammation in vascular remodeling is well-accepted although a number of questions on what initiates and maintains inflammatory state remains not completely understood. Here, we summarize the current knowledge on the contribution of TLR accessory molecules and downstream signaling in the course of cardiovascular remodeling.

## TLR Accessory Molecules That Act as Cofactors Required For Cell Surface Ligand Recognition and Delivery

### Myeloid Differentiation Factor 2—MD2

Myeloid differentiation factor 2 (MD2 also known as Ly96 or ESOP-1) is a small 18–25-kDa protein that binds to ectodomain of TLR4 and can also be secreted as a soluble molecule. TLR4-MD2 interactions are extremely important for proper signaling as no physiological role of TLR4 has been demonstrated in the absence of MD2. MD2 is responsible for LPS binding, TLR4 glycosylation and cell trafficking (63). LPS interacts with a hydrophobic residue in MD2 and directly bridges the two copies of the TLR4-MD2 multimer. The crystal structure of TLR4-MD2-LPS complex demonstrates how five of the six lipid chains of LPS embedded inside the MD2 pocket whereas the remaining chain is exposed to the surface of MD2. Formation of MD2-LPS complexes are essential for bridging the two TLR4 molecules and propagation of intracellular signaling (64).

Even though MD2 is known to be an important modulator of innate immune system, our knowledge on its role in cardiovascular remodeling is still limited.

Number of studies investigated the role of MD2 as biomarker for several CVDs. The clinical study by Riad et al. assessed the predictive value of MD2 in dilated cardiomyopathy (DCM) (65). DCM is characterized by ventricular chamber enlargement and systolic dysfunction, with most cases being idiopathic. Furthermore, a limited number of studies suggests that DCM associated with a chronic inflammatory state (66). MD2 is highly expressed within the myocardium of DCM patients as well as in murine cardiac tissue suggesting that MD2 can have local cardiac regulation of inflammatory responses. *In vitro* stimulation of cardiomyocytes with MD2 showed dose-depended negative ionotropic effect as demonstrated by cardiomyocyte cell shortening. In 174 patients diagnosed with DCM elevated MD2 blood levels served as additional biomarker to the gold-standard NT-pro-BNP for mortality risk prediction (67, 68).

Genome-wide association study in 304 individuals undergoing coronary artery bypass graft surgery identified potential positive association between MD2 and postoperative atrial fibrillation (AF). The same study identified genetic polymorphisms in the MD2 gene that were associated with decreased risk for postoperative AF (69). Further randomized trials should confirm the role of MD2 as a useful diagnostic and/or therapeutic target in DCM and postoperative AF.

Interesting data on the role of MD2 in atherosclerosis-driven cardiovascular diseases comes from a recent study by Chen et al. Here, the authors investigated to what extend MD2 participates in ox-LDL-induced TLR4 activation during atherogenesis. MD2 was highly present in atherosclerotic lesions from ApoE^−/−^ mice and in peripheral blood mononuclear cells (PBMC) from patients with coronary artery disease. In monocytes and macrophages MD2 is essential for ox-LDL-induced TLR4 dimerization, downstream activation of NF-kB and subsequent production of proinflammatory cytokines. Deficiency of MD2 reduced atherosclerotic plaques through reduced lesional macrophage content and expression of inflammatory cytokines (70).

Besides circulating immune cells, MD2 has local expression within vasculature including SMCs and endothelial cells, where it may regulate cell phenotypic switching (71, 72).

In an angiotensin II (Ang II) induced pathological model of aneurism formation, deficiency of MD2 resulted in the phenotypic switch of SMCs toward a proliferative phenotype and increased fibrosis (71).

Besides its well-known effect on arterial blood pressure, Ang II can directly induce cardiac remodeling through induction of TLR4 mediated cardiac inflammation (73). Han et al. demonstrated that MD2 mediates Ang II induced cardiac remodeling by direct binding to Ang II and the subsequent activation of the TLR4/MyD88/NF-kB signaling cascade (72). Moreover, in Ang II induced aortic remodeling MD2 was identified as a critical point in the Ang II induced endothelial-to-mesenchymal transition (EndMT) – an important mechanism of pathological vascular and cardiac remodeling. Pharmacological inhibition of MD2 reduced Ang II-induced EndMT changes, including increased levels of endothelial marker VE-cadherin, and reduction of mesenchymal markers alpha smooth muscle actin (α-SMA) and vimentin.

In obesity induced cardiac remodeling, both pharmacological inhibition and genetic deletion of MD2 resulted in attenuated cardiac inflammation and fibrosis via reduction of JNK, ERK and NF-kB signaling and reduced expression of cell adhesion molecules ICAM-1, VCAM-1, and CD68 (74).

Within the cardiovascular system MD2 was shown to interact with number of endogenous molecules that are released in response to stress, including free fatty acids (75, 76). In diabetic cardiomyopathy, advanced glycation end products (AGEs) bind to MD2 resulting in TLR4 activation and myocardial injury (77). Interestingly Huang et al. demonstrated the role of MD2 in vascular oxidative stress via SIRT1/MAPKs and reactive oxygen species (ROS) generation.

Taken together MD2-mediated chronic inflammation occurs in diverse cells and MD2 deficiency and pharmacological inhibition may alter a number of parallel pathways in vascular tissues (78). Promising results on the therapeutic benefits of MD2 inhibition comes from a recent study by Fang et al. Treatment with a small peptide Tat-CIRP which can pass through the blood brain barrier and competitively bind to MD2 was shown to induce long-lasting neuroprotection against ischemic and hemorrhagic stroke in rodents and non-human primates (79).

## LPS-Binding Protein—LBP

LPS-binding protein (LBP) is a 53 kDa protein that facilitates delivery of LPS to membrane bound and soluble CD14 to induce TLR4 signal transduction (80). Even though the crystal structure of LBP has not been reported, emerging state-of-the-art computer tools that utilize artificial intelligence allow to predict protein's 3D structure from its amino acid sequence that would help to foster our understanding of molecular interactions between LBP-LPS in the near future^1^.

Besides LPS, LBP can bind other PAMPs derived from gram-negative and gram-positive bacteria including lipopeptides and peptidoglycans. In addition to TLR4, upon ligand binding LPB can also activate other TLRs, including TLR1, TLR2, and TLR6 (81).

Recent attention to the contribution of microbiome in CVD supports the notion of endogenous PAMPs act as drivers of systemic CVD. A state of dysbiosis triggers production of endogenous PAMPs by the gut microbiota which in turn activates LBP-dependent low grade systemic inflammation. In this light proinflammatory action of LBP might contribute to vascular remodeling and development of cardiovascular complications suggesting its role as potential biomarker (82).

In two consecutive studies the LBP concentration was significantly elevated in patients with coronary artery disease and was associated with all cause and cardiovascular mortality (83, 84). In type 2 diabetes, increased levels of LBP correlated to diabetic retinopathy and arterial stiffness suggesting its role in the activation of local inflammatory response within the vasculature (85, 86).

In an exploratory study on 72 individuals LBP was reversely associated with CVD risk in older adults (87). Potentially counterintuitive, higher levels of cholesterol in elderly coupled with higher LBP may promote faster clearance of bacterial toxins from the circulation resulting in reduction of systemic inflammation (88).

Traditionally, LBP promotes TLR4 signaling shuttling to CD14 to interact with LPS. An elegant study by Han et al. explored the opposite effect of LBP on TLR4 cascade in the presence of intestine specific form of high density lipoprotein (HDL)—HDL3 (89). LBP was required to inhibit LPS-TLR4 signaling on liver macrophages via interaction with HDL3 particles. In this way, HDL3 interacts with LBP to mask LPS from detection by TLR4 signaling platform, resulting in an anti-inflammatory and anti-fibrotic mode of action. This finding might be relevant in cardiovascular disease as HDL3 levels correlate with better health outcomes (90).

These studies clearly indicate that there is a link between the accessory molecule LBP and CVD, however, more studies are needed to elucidate the role of LBP in various comorbidities and risk statuses.

## Cluster of Differentiation 36—CD36

CD36 is an 88 kDa membrane glycoprotein that belongs to the class B family of scavenger receptors. It is expressed on various cell types including monocytes/macrophages, platelets, dendritic cells, microglia, cardiovascular cells and adipocytes (91). Recent studies demonstrated that CD36 is involved in inflammation, angiogenesis, lipid metabolism and atherosclerosis progression. In CVD, CD36 is largely known as the receptor for oxLDL that accounts for 60 to 70% of cholesterol ester accumulation in macrophages. Solid number of literature on the role of CD36 in lipid trafficking and atherogenesis are available, see this excellent review (92), but will not be discussed here.

CD36 can interact with number of exogenous and endogenous ligands to facilitate downstream TLRs signaling. Recent studies revealed that CD36 induces the assembly of the TLR4/6 and TLR2/6 heterodimers underlining its role as TLR accessory molecule (93). Mediation of TLR4-TLR6 heterodimerization occurs from the C-terminus of CD36 within the cell. Point mutation at tyrosine 463 of CD36 resulted in inability of TLR4-TLR6 dimerization and NF-κB activation in response to oxLDL. In addition, a functional study reported the importance of CD36 interaction with Lyn kinase to assure TLR4 and/or TLR6 phosphorylation, TLR4-TLR6 association and signal transduction (93).

Interaction with microbial PAMPs mainly diacylglycerides LTA and R-MALP2 enhances TLR2-TLR6 mediated immune response (94) whereas binding to DAMPs including oxLDL, amyloid-b fibrils and apoptotic cells mediate TLR4-TLR6 inflammatory responses (93, 95).

In a hyperlipidemic ApoE^−/−^ model of atherosclerosis CD36 was shown to trigger TLR4-TLR6 dependent accumulation of TRIF-dependent chemokine RANTES and an overall increase in ROS production by macrophages. Such response amplifies oxidative stress in the artery wall, DAMPs generation and chronic macrophage activation (93).

Despite its apparent detrimental role in ischemic damage through activation of inflammation and ROS, CD36 may also exert a beneficial role during post-stroke and post-MI resolution phase of inflammation by mediating phagocytosis (96, 97). Interestingly, in the absence of elevated circulating lipids cardiomyocyte-specific deletion of CD36 accelerated the progression of pressure overload-induced cardiac hypertrophy to cardiac dysfunction (98). Contrary, CD36 can promote neovascularization and scar formation worsening post-stroke recovery (99, 100).

Clearly, CD36 is a multifunctional receptor that may play different roles in the CVD pathogenesis/repair. Further mechanistical studies on the role of CD36 as TLR accessory molecule will help to determine its potential as a therapeutic target.

## Cluster of Differentiation CD14—CD14

CD14 is a pattern recognition receptor that has long been known as co-receptor for several TLRs. It is a 375 amino acid LRR-containing glycoprotein that can be secreted into the serum as a soluble molecule (sCD14) or expressed as a glycosylphosphatidylinositol (GPI)-linked membrane bound protein on the surface of cells (mCD14) (101). mCD14 is highly expressed on myeloid cells, whereas sCD14 is present in different body fluids to transduce LPS-responsiveness to cells not expressing CD14 (102, 103). Before the discovery of TLR in 1997 (104), CD14 was known as a cell differentiation marker for human monocytes and classical dendritic cells (105, 106). The crystal structure demonstrates CD14 to form a dimer with hydrophobic pocket located at its N-terminus that is essential for recognition of LPS and other microbial peptides. Formation of CD14-LPS complex is important to enhance the detection of LPS by the TLR4–MD2 complex by monomerizing LPS before its presentation to TLR4–MD2 (107).

CD14 has a wide range of exogenous and endogenous ligands, including bacterial PAMPs, heat shock proteins, phospholipids and amyloid. CD14 is involved in ligand delivery and functions as an adaptor and coreceptor of TLRs to increase ligand affinity to TLR and augment signal transduction (108–110). CD14, together with TLR4 and MD-2, forms the multi-receptor complex that recognizes LPS on the cell membrane resulting in activation of MyD88-dependent signaling (111). Upon low doses of LPS CD14 allows the activation of the intracellular TLR4-TRAM-TRIF pathway resulting in TLR4 internalization and endocytosis (112). As a coreceptor CD14 is necessary for the TLR7- and TLR9-dependent induction of proinflammatory cytokines *in vitro* and for TLR9-dependent innate immune responses in mice (113, 114).

Uncontrolled inflammation with TLR overactivation on CD14^+^ monocytes has long been recognized as a driving mechanism for atherosclerotic disease (115, 116). Population of intermediate monocytes with increased mCD14 and CD16 can independently predict cardiovascular events in patients with coronary artery disease (117, 118). CD14 is also known to regulate function of endothelial and smooth muscle cells. The interaction of LPS with CD14 on the surface of endothelial cells results in expression of proinflammatory cytokines and adhesion molecules (119). Local expression of CD14 by human coronary artery smooth muscle cells can potentially increase tissue sensitivity to pro-atherogenic risk factors (120).

Upregulation of CD14 in adventitial macrophages in a murine model of abdominal aortic aneurism (AAA) and both locally and systemically in human AAA underscore its role in the pathogenesis of AAA (121).

In the central nervous system CD14 deficiency causes attenuated monocyte influx to the brain and aggravation of tissue injury after ischemic stroke (122).

sCD14 has gained interest as a risk factor for different CVD. Important to mention are racial difference in sCD14 levels. Elevated plasma sCD14 was an independent risk factor for heart failure, coronary heart disease and stroke in cohorts of African Americans whereas was not linked to increase incidence of CVD in Caucasians (123–125). Activation of endothelial cells through sCD14 results in upregulation of adhesion molecules and procoagulant activity (108).

A number of recent studies unraveled the contribution of disturbed intestinal barrier toward chronic low-grade endotoxemia. Bacterial LPS binds and stimulates systemic secretion of CD14 which is required for propagation of TLR signaling. CD14-LPS complex is implicated in LPS-induced myocardial dysfunction (126).

In summary, as mCD14 but also as sCD14, CD14 exerts significant impacts in the pathogenesis of cardiovascular diseases. It can both promote and diminish TLR signaling which makes it an important regulator of the inflammatory response.

## TLR4 Interactor With Leucine-Rich Repeats – TRIL

TRIL (TLR4 interactor with leucine-rich repeats) is a 811 amino acid leucine-rich repeat protein that plays an important role in TLR3 and TLR4 signaling. TRIL is primarily expressed in the brain where it interacts with LPS or poly(I:C) ligands to activate TLR4 and TLR3 signaling, respectively (127, 128).

TRIL induction helps to prolong LPS signaling and is structurally similar to CD14 which makes it a functional homolog of CD14. As LRR- containing protein without signaling domain TRIL could also have a role similar to RP105 positively or negatively regulating TLR4 signaling in a cell-type-dependent manner. Whether it can bind to MD1 and/or MD2 as well as its role beyond the brain is not defined yet.

Some preliminary data indicates that TRIL might be required to control lipid-based ligands for TLR2 signaling.

Recently Jia et al. were the first to demonstrate a role of TRIL-TLR4 signaling in the progression of spinal cord ischemia reperfusion injury (129). Due to its localized expression to the brain contribution of TRIL to systemic CVD remains obscure.

## Non-Classical Accessory Molecules With TLR Signaling and Crosstalk Function

### Radioprotective 105—RP105

RP105 (radioprotective 105, CD180) is a TLR-like accessory molecule that has a striking structural similarity to TLR4 with LLR domain and association with MD1, an MD2 homolog. Unlike the TLRs, however, RP105 lacks an intracellular signaling TIR domain. The crystal structure of RP105-MD1 bound to LPS shares a similar overall architecture to its homolog TLR4–MD2. Interestingly, assembly of RP105-MD1 homodimer occurs in a head-to-head orientation with N-termini interacting in the middle which is different from TLR4-MD2 complex with the tail-to-tail configuration of C-terminal cytoplasmic signaling domain. Such unique mode of assembly in RP105–MD1 suggesting a potential molecular mechanism for regulating LPS responses and regulation of TLR4-MD2 signaling complex (130). Depending on the cell type RP105-MD1 exerts dual regulatory activity on TLR-regulated inflammatory response. On B-cells, where it was originally discovered, it stimulates cell proliferation and antibody production (131, 132). On myeloid cells, including monocytes, macrophages and dendritic cells RP105-MD1 complex acts mainly as TLR4 antagonist. Importantly, RP105-MD1 is also expressed locally within cells of the CV system, including arterial and venous SMCs, endothelial cells and cardiomyocytes (133). A number of studies indicate a role for RP105 in several CVD pathologies as both physiological inhibitor and agonist of TLR4 signaling.

RP105 was shown to play an important role in IRI. In a model of hind limb ischemia RP105 deficiency resulted in an uncontrolled inflammatory response, impaired blood flow recovery and reduced arteriogenesis. These outcomes were linked with premature systemic activation of pro-inflammatory monocytes (Ly6C^hi^) in RP105 knockout animals that resulted in accumulation of Ly6C^hi^ monocytes in the bone marrow and spleen rather than in the ischemic tissues where the reparative response was needed (134).

Several studies explored the role of RP105 in myocardial infarction. In a model of a short-term myocardial ischemia-reperfusion injury (MIRI) adenoviral delivery of RP105 protected myocardium against IRI via inhibition of TLR4/TLR2 inflammatory cascade and regulation of cardiomyocyte apoptosis and autophagy via Bcl-2/ Beclin1 complex (135–137). *In vitro*, overexpression of RP105 was effective to protect cardiac microvascular endothelial cells against hypoxia induced reoxygenation injury (133). As expected, knockdown of RP105 resulted in increased myocardial infarction size during MIRI that was associated with TLR2/4 activation and increase in myocardial cell apoptosis (138).

Louwe et al. examined the effects of RP105 on cardiac function post-MI using permanent ligation of the left anterior descending coronary artery model that closely mimics MI in humans. RP105 deficiency hampered repair processes and resulted in the right shifted PV-loop similar to what is seen in dilated cardiomyopathy. Interestingly, even though RP105 deficiency resulted in enhanced inflammatory status and more pronounced cardiac dilation, there was no difference in the infract size between RP105^−/−^ and WT animals (139).

RP105 was shown to contribute to vascular remodeling in vein graft disease and failure of hemodialysis vascular access conduit. Vein graft disease progression is linked to intimal hyperplasia, inflammation and superimposed atherosclerosis development. It is known that TLR4 signaling plays an important role in vein graft disease progression (56). In a study by Wezel et al. RP105 deletion exacerbated vein graft disease in the presence of hypercholesterolemia. Increase in lesional CCL2 expression resulted in macrophage and mast cell accumulation accompanied by lesion destabilization and intraplaque hemorrhage (140).

Similar to vein graft disease, arteriovenous fistula failure is associated with increased inflammation and intimal hyperplasia. Impaired outward remodeling is another important parameter of successful vascular remodeling assuring sufficient blood flow during dialysis treatment. RP105 deficiency resulted in an impaired outward remodeling of murine arteriovenous fistula. Interestingly, the importance of RP105 in the balance between pro-inflammatory and regenerative response in macrophages and SMCs was demonstrated (141). Specifically, accumulation of anti-inflammatory macrophages in vascular lesions from RP105 deficient mice and decrease in proliferation and migration of *in vitro* cultured venous and arterial SMCs, respectively.

Therapeutic delivery of soluble RP105 by electroporation mediated gene transfer was shown to be an effective strategy to dampen vascular remodeling and intimal hyperplasia in a mouse model of post-interventional restenosis (142), but only when solRP105 was co-expressed with MD1.

Contrary, in atherosclerosis RP105 appears to play the role of a negative regulator. RNA levels of RP105 are upregulated early during atherogenesis. Deletion of RP105 was linked to decrease in plaque development and CCR2 dependent inhibition of monocyte influx (143). Wezel et al. very elegantly showed an altered migratory capacity of monocytes upon deletion of RP105, and that *in vitro* stimulation of monocytes with LPS induced a downregulation of CCR2, a chemokine receptor crucially involved in monocyte influx to atherosclerotic lesions, which was more pronounced in RP105^−/−^ monocytes. In a different model of atherosclerosis, bone marrow transplantation from RP105^−/−^ mice into hyperlipidemic LDLR^−/−^ recipients resulted in plaque size decrease. Such effect was explained by reduction in activation and proliferation of proinflammatory subset of B-cells and diminished production of immunoglobulin IgG2c (144).

From the literature it is clear that RP105 is involved in the complex regulation of vascular remodeling and control of TLR4-mediated inflammatory response in different cardio-vascular pathologies. To design a successful therapeutic strategy targeting TLR4/RP105 axis cell specific targeting and time of the application should be taken into consideration in view of these complex pathophysiological roles.

### Myeloid Differentiation 1—MD1

Myeloid differentiation 1 (MD1, also known as lymphocyte antigen Ly86) is a secreted glycoprotein that interacts with RP105 to assure sufficient RP105 cell surface expression whereas MD1 acts as an MD2 homolog (145). MD1 has a similar structure to MD2 with a hydrophobic cavity that accommodates LPS or related microbial peptides (146). As we previously described, the RP105-MD1 complex plays an important role in TLR4 signaling. Expression of MD1 is not limited to the cells of the immune system but is highly expressed within cardiac tissue^2^. A number of studies elaborated on the role of MD1 in pressure induced cardiac remodeling and post IRI myocardial adaptation amongst others, hypertrophy, fibrosis, arrythmias and heart failure.

Cardiac hypertrophy is a compensatory mechanism in response to biomechanical wall stress wall that is associated with increases in cardiomyocyte size, increased ***extracellular matrix*** synthesis and a higher organization of sarcomere (147). Even though pathways that promote hypertrophic response are well-defined, little is known on key molecular players underlying these pathways that can serve as effective therapeutic targets. Xiong et al. were the first to demonstrate the importance of MD1 in pathological cardiac remodeling (148). Constitutive cardiac overexpression of MD1 in mice had a prominent effect against cardiac hypertrophy and fibrosis via inhibition of TLR4 downstream signaling molecules MEK-ERK1/2 and NF-κB, whereas loss of MD1 caused chronic pressure overload induced cardiac remodeling. Hyperactivation of TLR4 signaling in the absence of MD1 triggers Ca^2+^/calmodulin-dependent protein kinase II (CAMKII) signaling, resulting in alteration of Ca^2+^ handling and K^+^ and Na^+^ channels in stressed myocardial tissues (149). Consistent with previous findings, in a model of myocardial infarction MD1 depletion resulted in elevation of MI-induced fibrosis, inflammation and electrical remodeling via upregulation of TLR4/CaMKII signaling that was linked to increased vulnerability to ventricular arrhythmias (150). The link between inflammatory TLR4 signaling and regulation of ion channels in LV structural and electrical remodeling further support importance of accessory molecule MD1 as potential therapeutic strategy in heart failure.

Interestingly, in a model of post-interventional vascular remodeling overexpression of MD1 alone did not have any effect on vascular remodeling whereas simultaneous expression of soluble RP105 and MD1 resulted in a significant reduction in intimal hyperplasia (134). In line, Divanovic et al. showed that RP105 can only act as inhibitor for TLR4 signaling if MD1 is sufficiently present (151). Similar to MD2 dependent TLR4 surface expression, RP105 surface expression was shown to be dependent on MD1 (145).

Expression of MD1 was reported to be decreased in hearts from obese patients. Obesity is a known risk factor in metabolic syndrome development that is associated with cardiac remodeling and impairment of left ventricular function. When mimicking obesity in a mouse model, deletion of MD1 aggravated high-fat diet-fed induced maladaptive left ventricular hypertrophy *via* TLR4/MyD88/CaMKII (152) and TLR4/NF-kB signaling (153). The role of MD1 seems to be of particular importance in obesity-related structural and electrical remodeling as it triggers not only ventricular but also atrial remodeling via the earlier described TLR4/MyD88/CaMKII signaling pathway (154).

A recent study demonstrated the importance of MD1 in myocardial ischemia-reperfusion injury and IRI related arrhythmia (155). Loss of MD1 led to a larger infarct size, increase in pro-inflammatory TNF, IL1β, and IL6 plasma levels, induction of myocardial apoptosis and accumulation of neutrophils and macrophages. In a concomitant study MD1 overexpression protected myocardial function against high-fat induced hypertrophic cardiomyopathy, further supporting the importance of TLR4 inflammatory signaling in the mechanism of myocardial injury and therapeutic potential of MD1 in cardiac remodeling (156).

### Dectin-1

Dectin-1 is a pattern recognition receptor that belongs to the class of C-type lectin receptors and is mainly expressed on activated myeloid cells (157). When bound to its ligand, β-glucans, dectin-1 initiates recruitment and phosphorylation of spleen tyrosine kinase (SYK), thereby activating the NF-κB dependent inflammatory cascade. Traditionally, dectin-1 has been associated with the recognition of fungi, but recent discoveries underlined its role in non-infectious diseases including those related to the cardiovascular system. Dectin-1 aggravates cardiac remodeling after myocardial infarction and worsens inflammatory response after ischemic stroke (158, 159). In patients, increase in circulating dectin-1^+^ monocytes correlates with the severity of cardiac dysfunction (160).

One study indicates that there is a crosstalk between dectin-1 and TLR2 signaling in response to fungal pathogens suggesting that dectin-1 is involved in crosstalk of TLR-dependent signaling pathways. Contribution of dectin-1 and its therapeutic potential in the context of TLRs mediated responses in the pathogenesis of cardiovascular requires further investigation (161).

### Vitronectin

Vitronectin is a 75 kDa glycoprotein that is present in plasma, ECM, and in alpha-granules of blood platelets. It has a major impact on cell adhesion, migration and vascular remodeling via interaction with integrin receptors. Similar to dectin-1, via interaction with its receptor integrin beta3 vitronectin was shown to enhance TLR2-mediated responses to microbial lipopeptides (162). In addition, vitronectin was reported to enhance TLR4 mediated signaling by recruitment of the adaptor protein TIRAP to the plasma membrane (163).

Vitronectin plays role in vascular remodeling via activation of adhesion and migration of SMCs contributing to intimal hyperplasia (164) and it is highly expressed in atherosclerotic plaques (165). Several integrins expressed on vascular SMCs and platelets, including vitronectin receptor αvβ3, recognize the Arg-Gly-Asp (RGD) sequence present on many adhesion molecules, to participate in the cell adhesion to vitronectin and migration of the cells toward fibronectin, laminin, and collagen types I and IV. Thus, SMCs adhesion to vitronectin can be inhibited by RGD-containing peptides to prevent formation of intimal hyperplasia (166). In a hamster model of intimal hyperplasia induced by surgical damage of the carotid arteries and continued administration of G4120, a cyclic RGD-containing peptide, reduced the platelet activation and the SMCs content, preventing intimal hyperplasia formation as compared to untreated animals (167). In a rat model of arterial injury caused by balloon angioplasty, administration of abciximab—monoclonal antibodies involved in inhibition of integrin signaling, prevented formation of intimal hyperplasia. Abciximab interacts with αIIbβ3 (glycoprotein IIb/IIIa complex) integrin on platelets that inhibits platelets adhesion in injured vessels and it can also bind to αvβ3—vitronectin receptor present of SMCs hindering cell migration and proliferation (168).

Vitronectin contributes to regulation of vascular homeostasis at sites of vascular injury. It stabilizes PAI-1—a central physiological inhibitor of plasminogen activation (169, 170) and can bind to platelet glycoproteins mediating platelet adhesion and aggregation (171).

Interestingly, elevated circulating levels of vitronectin correlated with female specific increase in inflammation after ischemic stroke in mice which points toward its prognostic value for stroke outcomes in women (172).

Even though vitronectin was shown to contribute to vascular remodeling, the mechanistic link between vitronectin and TLR signaling is not yet well defined. Clearly, further studies are required to elucidate the mechanism of vitronectin-TLR mediated vascular remodeling.

## SARS-CoV-2 AND TLR Signaling

In light of the current pandemic of coronavirus disease 2019 (COVID-19) caused by severe acute respiratory syndrome coronavirus 2 (SARS-CoV-2) for the scope of this review it is important to mention vascular complications that are linked to TLRs immune response (173). SARS-CoV-2 induces release of proinflammatory cytokines and activation of procoagulant factors that activate coagulation cascades leading to thrombosis, rupture of atherosclerotic plaques and ischemic events (174, 175). Clinical studies demonstrated that hypercoagulability and vascular complications are utmost important predictors of disease outcomes (174, 176, 177). Recent work by Zheng et al., demonstrated contribution of TLR signaling in response to SARS-CoV-2 that results in proinflammatory cytokine production and disturbed immune response as seen in patients with severe form of COVID-19 (178). Interestingly, increase in mRNA expression levels of adaptor protein Myd88 as well as various TLRs that signal through it were positively correlated with disease severity. Further mechanistic study in Myd88^−/−^ mice infected with mouse hepatitis virus (MHV), a laboratory analog for SARS-CoV-2 resulted in reduction in TNF as compared to WT controls. Subsequent *in vitro* experiments with SARS-CoV-2 infected peripheral blood mononuclear cells (PBMCs) demonstrated that inhibition of TLR2, but not TLR4, resulted in downregulation of cytokine and chemokine production. This study elegantly demonstrates importance of TLR2 and Myd88 in sensing SARS-CoV-2 by triggering inflammatory response and release of proinflammatory cytokines such as TNF and IFN-γ. Therapeutic targeting of the immune response such as inhibition of TLR2 might be a promising therapeutic target for mitigating COVID-19 severity. Besides induction of pro-inflammatory signaling, TLRs are also programmed to activate negative feedback loops to exert anti-inflammatory and tissue repair signals. COVID-19 disease is highly associated with the development of the cytokine storm due to uncontrolled TLR response – a devastating inflammatory reaction leading to multi-organ failure and death. It is not known how the TLR-induced negative regulatory mechanisms fail to inhibit exaggerated activation of TLRs and associated cytokine storm. Administration of MD2-TLR4 antagonist Eritoran has shown effective downregulation of TLR4 in animal model of sepsis (179), but did not proof to be effective in patients (180). During acute influenza virus infection Eritoran has successfully targeted the cytokine storm in experimental studies (181). The protective action of Eritoran also involves CD14 and TLR2. The Eritoran directly binds to the CD14 preventing the ligand binding to MD2 or lymphocyte antigen 96 (181). Hence, further studies on the role of TLR accessory molecules are relevant to design effective therapeutics to target inflammatory diseases, including cytokine storm associated with SARS-CoV2 infection-induced severe COVID-19.

## Concluding Remarks and Therapeutic Perspectives

**Figure 2:**
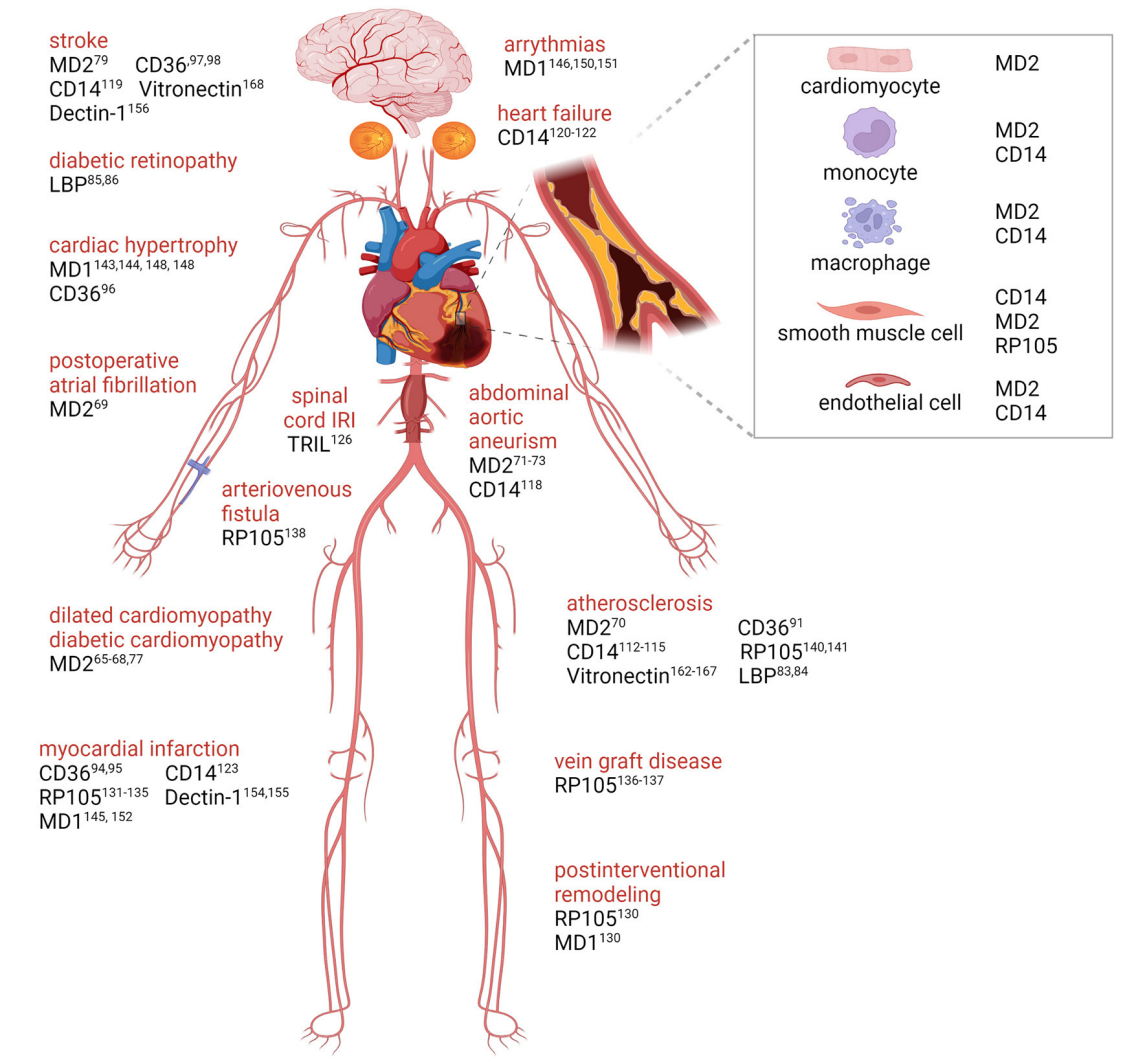
TLRs accessory molecules in cardiovascular disease. Graphical overview with supporting literature references underlying the role of accessory molecules for membrane TLRs that are involved in cardiovascular disease progression. Created with BioRender.com.

Not even three decades have passed since the discovery of TLR, nonetheless this field has rapidly evolved and its importance was underscored by the Nobel Prize in Medicine in 2011. TLR can recognize a broad repertoire of PAMPs and DAMPs yet contain structurally conserved ectodomains. Accessory molecules provide an important tool to ligand discrimination and receptor signaling to assure proper TLR specificity, signal transduction and tissue response. In CVD, inflammation plays an important role in both disease progression and resolution as has been showing elegantly the successes of the CANTOS trial (17) and the COLCOT and LoDoCo2 trials (182, 183). Single cell analysis of various cardiovascular diseases further confirmed the involvement of downstream TLR signaling inflammatory cytokines further illustrating the importance of these pathways. In this review we reported on the complex regulatory mechanisms that are involved in the regulation of the TLR signaling pathways triggered by the broad range of ligands, both exogenous and endogenous. As indicated the various accessory molecules are key regulators in the disease specific or cell type specific responses of TLR activation (Figure 2). This of course suggests a new line of potential therapeutic approaches based on manipulating TLRs and their downstream signaling as has been described for the use of soluble coreceptors like soluble RP105 (142). Overexpression of the soluble RP105 was shown to dampen the TLR4 mediated inflammatory response in vascular remodeling, by acting as a decoy receptor of the TLR4 binding DAMPS. However, these experiments directly demonstrated the complexity of such an approach since solRP105 was only functionally active when co-expressed with the cofactor MD1. The latter is required for the stability of the solRP105 protein (142). This is illustrative for the lack of knowledge in this field to translate these findings toward actual therapeutic application. Better understanding of the regulation of TLR signaling via accessory molecules in vascular and cardiac remodeling could help to develop new therapies aiming at guiding beneficial TLR response.

## Author Contributions

TB, JK, MV, and PQ conceptualized and formulated review aims. TB and MV developed methodology, prepared original draft, and collected data and evidence. JK and PQ performed reviewing and editing. TB visualization. PQ and MV supervised the research. All authors discussed the results and contributed to the final manuscript.

## Funding

This work was supported by Partners of Regenerative Medicine Crossing Borders (www.regmedxb.com) and powered by Health~Holland, Top Sector Life Sciences & Health.

## Conflict of Interest

The authors declare that the research was conducted in the absence of any commercial or financial relationships that could be construed as a potential conflict of interest.

## Publisher's Note

All claims expressed in this article are solely those of the authors and do not necessarily represent those of their affiliated organizations, or those of the publisher, the editors and the reviewers. Any product that may be evaluated in this article, or claim that may be made by its manufacturer, is not guaranteed or endorsed by the publisher.

## Footnotes

^1^Available online at: https://alphafold.ebi.ac.uk/.

^2^Available online at: https://www.proteinatlas.org/. (2003).

## Abbreviations

AAA: abdominal aortic aneurism
AF: atrial fibrillation
AGEs: advanced glycation end products
ALRs: Absent in melanoma-2 (AIM2)-like receptors
AngII: angiotensin II
CAD: coronary artery disease
CLRs: C-type lectin receptors
CVD: Cardiovascular disease
DAMPs: Damage associated molecular patterns
DCM: dilated cardiomyopathy
ECM: Extracellular matrix
EndMT: endothelial-to-mesenchymal transition
ER: Endoplasmic reticulum
FFA: free fatty acids
HMGB-1: High Mobility Group Box-1
HSPs: Heat Shock Proteins
IFN-I: Type I interferon
IRFs: Interferon regulatory factors
IRI: Ischemia reperfusion injury
LBP: LPS-binding protein
LDL: low density lipoprotein
LPS: Lipopolysaccharide
LRRs: Leucine-rich repeats
MD1: Myeloid differentiation factor 1
MD2: Myeloid differentiation factor 2
MI: Myocardial infarction
MIRI: myocardial ischemia-reperfusion injury
MyD88: Myeloid differentiation primary response protein 88
NLRs: Nucleotide-binding oligomerization domain-like receptors
NT-proBNP: N-terminal pro b-type natriuretic peptide
PAMPs: Pathogen-associated molecular patterns
PBMC: peripheral blood mononuclear cells
RLRs: Retinoic acid-inducible gene-I-like receptors
ROS: reactive oxygen species
RP105: Radioprotective protein 105
PRRs: Pattern recognition receptors
SMCs: Smooth muscle cells
TIR: Toll/interleukin-1 receptor-like
TLRs: Toll-like receptors
TRIL: TLR4 interactor with leucine-rich repeats.
